# Mobile Elements in Ray-Finned Fish Genomes

**DOI:** 10.3390/life10100221

**Published:** 2020-09-25

**Authors:** Federica Carducci, Marco Barucca, Adriana Canapa, Elisa Carotti, Maria Assunta Biscotti

**Affiliations:** Dipartimento di Scienze della Vita e dell’Ambiente, Università Politecnica delle Marche, 60131 Ancona, Italy; f.carducci@univpm.it (F.C.); m.barucca@univpm.it (M.B.); a.canapa@univpm.it (A.C.); e.carotti@pm.univpm.it (E.C.)

**Keywords:** ray-finned fishes, transposable elements, genome evolution

## Abstract

Ray-finned fishes (Actinopterygii) are a very diverse group of vertebrates, encompassing species adapted to live in freshwater and marine environments, from the deep sea to high mountain streams. Genome sequencing offers a genetic resource for investigating the molecular bases of this phenotypic diversity and these adaptations to various habitats. The wide range of genome sizes observed in fishes is due to the role of transposable elements (TEs), which are powerful drivers of species diversity. Analyses performed to date provide evidence that class II DNA transposons are the most abundant component in most fish genomes and that compared to other vertebrate genomes, many TE superfamilies are present in actinopterygians. Moreover, specific TEs have been reported in ray-finned fishes as a possible result of an intricate relationship between TE evolution and the environment. The data summarized here underline the biological interest in Actinopterygii as a model group to investigate the mechanisms responsible for the high biodiversity observed in this taxon.

## 1. Introduction

**Figure 1:**
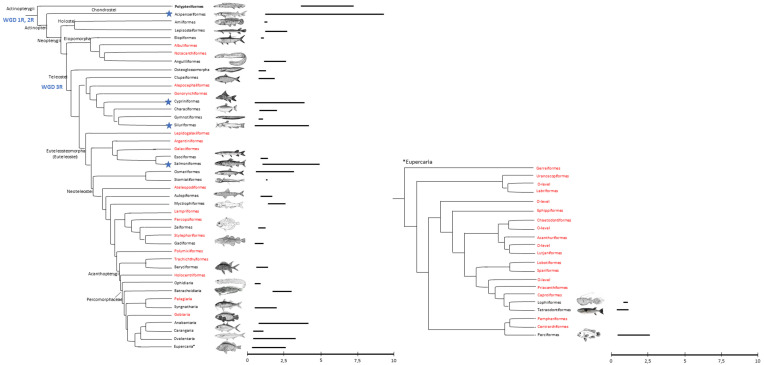
On the left: cladogram showing the relationships of bony fishes (modified from Betancur-R et al. [7]), with the relative DNA content from the genome size database [6]. The teleost orders lacking genome size information are shown in red. Whole-genome duplications (WGDs) are shown in blue: WGD 1R and 2R occurred in the common ancestor of vertebrates; WGD 3R occurred in the common ancestor of teleosts. The blue stars indicate taxa that underwent further independent WGD events. On the right: a separated cladogram of the Eupercaria clade with its relative nuclear DNA content. Order-level incertae sedis (O level in this figure) includes families awaiting evidence to clarify their phylogenetic status [7].

Actinopterygii is one of the most diverse groups of vertebrates, with over 30,000 species [1]. This taxonomic group includes Polypteriformes, Acipenseriformes, Holostei, and Teleostei. The order Polypteriformes comprises a unique family, Polypteridae, with two extant genera, *Erpetoichthys* (including only one species) and *Polypterus* (including eleven species). These fish appeared during the Cretaceous and inhabit equatorial and subequatorial freshwater areas in Africa. Several molecular phylogenetic analyses have shown that bichirs are a basal lineage of the ray-finned fishes [2,3,4,5]. Data about genome size are available for only four species, with values ranging from 3.69 to 7.25 pg/N [6] (Figure 1), while entire genomic DNA sequencing has been performed for only *Erpetoichthys calabaricus* (Ensembl release 99—January 2020). The order Acipenseriformes includes two families: Acipenseridae (sturgeons), with four extant genera (*Acipenser*, *Huso*, *Scaphirhynchus*, and *Pseudoscaphirhynchus*), contains 24 species, and Polyodontidae (paddlefish), with two extant genera (*Polyodon* and *Psephurus*), contains three species. These fish are widely distributed in the rivers, lakes, and seas of Northern Hemisphere countries. Data from 17 species reveal that these taxa have a very wide range of genome sizes, spanning from 1.22 to 9.32 pg/N [6]. Moreover, the high value (9.32 pg/N) recorded in *Acipenser brevirostrum* is the highest value among ray-finned fishes (Figure 1).

Holostei and Teleostei are part of the Neopterygii, a group of fishes that appeared in the late Permian and were characterized by better swimming capabilities and feeding mechanisms that allowed them to colonize a wider range of habitats. The infraclass Holostei comprises two orders: Amiiformes (bowfins) has a single family, Amiidae, which includes only one living species, *Amia calva*; Lepisosteiformes also has a single family, Lepisosteidae (gars), including seven species subdivided into two genera, *Atractosteus* (three species) and *Lepisosteus* (four species). *Amia calva* is found in freshwater rivers and lakes of North America, while gars are distributed in fresh, brackish, and, sometimes, marine waters of North and Central America. Genome sizes are available for only three species of Holostei, with an average of 1.29 pg/N [6], and only the genome of *Lepisosteus oculatus* has been fully sequenced (Ensembl release 99—January 2020). Teleostei is the most successful group of ray-finned fishes, with more than 24,000 species subdivided into 72 orders [7]. Teleosts appeared in the Late Triassic, and their evolutionary radiation occurred during the Mesozoic and Cenozoic. They have adapted to aquatic environments worldwide, from salt to fresh waters, from cold to warm seas, and from high-elevation mountain lakes to extreme sea depths [8]. In the genome size database, more than 1800 teleost species are listed, with values ranging from 0.34 pg/N (the most compact genome among vertebrates, recorded in a species of the Tetraodontidae family) to 4.90 pg/N (in a species belonging to the Salmonidae) [6], and the genomes of more than 300 species have been sequenced (NCBI genome database).

Knowledge of genome composition and architecture is fundamental in the comprehension of the evolutionary processes responsible for fish radiation. The advent of high-throughput sequencing technologies and bioinformatics has provided a great amount of genomic data, which has been extremely useful for obtaining insights into the evolution of ray-finned fish genomes. Indeed, the identification of gene loss and duplication events, genomic arrangements, variation in base composition, and different selection pressures on specific genomic regions have been highlighted through comparative genomics [8]. Moreover, it is known that, as in other vertebrates, the genome of actinopterygians underwent two rounds of whole-genome duplication (WGD) [9,10,11]. A third event occurred in teleosts approximately 226–350 Mya, leading to duplicated genes that were probably responsible for the radiation of these clades [11,12,13]. A fourth round of whole-genome duplication occurred independently in Neotropical Corydoradinae catfishes between 54 and 66 Mya [14], in salmonids approximately 88–103 Mya [15,16] and in cyprinids, approximately 5.6–11.3 Mya [17]. Another interesting aspect of the fish genome is the GC content. On the basis of GC percentage, genomes can be divided into five isochores, i.e., regions longer than 300 kb, with a high degree of uniformity in guanine and cytosine: two light isochores, L1 with 34–36% and L2 with 37–40%, and three heavy isochores, H1 with 41–45%, H2 with 46–52%, and H3 with more than 53%. While the genomes of mammals and birds contain heavy isochores, resulting in GC heterogeneity, fish and amphibian genomes have isochores with low CG content and typically have two light isochores [18]. Excluding salmonids, a negative correlation between genome size and genomic GC% in fish has been reported [19]. In contrast, nonteleost gars possess an AT/GC compartmentalized genome [20], and their closest living relatives, the bowfin *Amia calva*, have a typical teleost-like AT/GC homogenous genome, despite being nonteleosts [19].

Finally, the presence of mobile elements in fish genomes most likely contribute to shaping their genomes, providing advantageous features that have allowed them to successfully adapt to different environments [21,22,23]. Given the ability to move throughout the genome, the impact of mobile elements on genome evolution is higher than commonly supposed, and several papers have recognized the role of these elements as one of the most powerful evolutionary tools [23]. On the basis of these premises, this review is focused on the importance of mobile genetic elements for the genomes of actinopterygian, one of the most diverse vertebrate groups (Table 1). 

## 2. Mobile Elements

Mobile elements are genetic elements capable of moving throughout the genome by a transposition mechanism. The effect of their movement can be deleterious for the host genome if they interrupt genes; in contrast, it can lead to advantageous innovations, creating new genes or regulatory sequences through a process called molecular domestication.

On the basis of their transposition intermediate, either RNA or DNA, mobile elements can be distinguished into two main classes, according to the classification proposed by Wicker and colleagues [153] (Figure 1).

**Figure 2:**
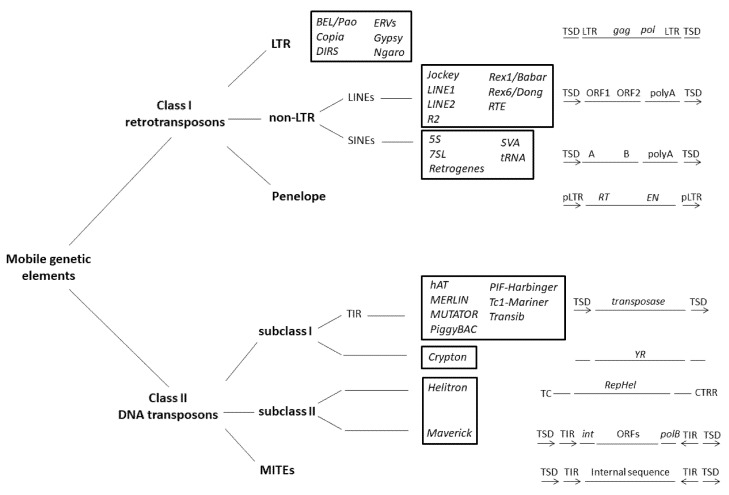
Mobile genetic element classification based on transposition mechanisms (according to Wicker et al. [153]). The main structural components of the elements are reported on the right (modified from Makalowsky et al. [155]).

Class I elements transpose via RNA intermediaries and are characterized by a *copy and paste* transposition mechanism. Their RNA intermediate is reverse-transcribed into its complementary DNA by a reverse transcriptase (RT) encoded by the mobile element. Reverse transcription is followed by reintegration into the host genome. Through the *copy and paste* mechanism of transposition, Class I elements are the main source of increased repetitive fractions, thereby having a major impact in large genomes [153,154,155]. Class I mobile elements are composed of long terminal repeat (LTR) and non-LTR subclasses. LTR retrotransposons are characterized by long terminal repeats that confer the ability to transpose. For exogenous retroviruses, LTR retrotransposons are structurally composed of *gag* and *pol* genes; *gag* genes encode viral structural particles and *pol* genes encode the whole retrotranscription machinery (*reverse transcriptase*, *ribonuclease H*, and *integrase*; Figure 2). In contrast to LTR retrotransposons, exogenous retroviruses possess the *env* gene, which encodes the viral envelope. However, traces of the *env* gene have been found in LTR retrotransposons [156]. *DIRS*, considered more complex LTR retroelements [37], are structurally characterized by a *tyrosine recombinase* (*YR*) instead of an *integrase* and by inverted terminal repeats. Long and short interspersed nuclear elements (LINEs and SINEs) are non-LTR retrotransposons. Of these, LINEs are autonomous retroelements constituted by two open reading frames (ORFs) and a poly A tail at the 3′ end. Generally, ORF2 encodes a reverse transcriptase and an endonuclease protein [153]. In contrast, SINEs are *RT*-lacking retroelements, and they need RT encoded by autonomous elements to transpose [157]. Finally, another group of Class I elements, Penelope retroelements, must be considered separately due to their very large diversity in terms of structural features. The common components are pseudo-LTRs (pLTRs), a *reverse transcriptase*, and an *endonuclease* [158,159].

Class II mobile elements use a DNA intermediate to transpose their genomic DNA copies into a novel chromosomal position [160,161] and can be divided into subclasses I and II. Subclass I consists of two main elements: TIR and *Crypton*. *TIRs* are autonomous elements characterized by terminal inverted repeats (TIRs) and a transposase through which transposition occurs via a *cut and paste* mechanism, in which both DNA strands are cleaved. The DNA transposons *hAT*, *Merlin*, *Mutator*, *PiggyBac*, *PIF*-*Harbinger*, *Tc1-Mariner*, and *Transib* can be found in this subclass. *Crypton* elements use a tyrosine recombinase (YR) in a transposition mechanism, probably involving recombination between a circular intermediate and the DNA target [37]. *Helitrons* and *Maverick* are the two major representative elements of subclass II. These DNA elements transpose via a *copy and paste* mechanism [153]. *Helitron* DNA transposons replicate using a rolling-circle mechanism and encode for replication initiation (Rep) and a DNA helicase (Hel) [162], while *Maverick* transposons encode for an integrase, an ORF, and polymerase B. For polymerase B, transposition involves a single-strand excision phase, extrachromosomal replication, and consequent reintegration into a new location [163]. Miniature Inverted Transposable Elements (MITEs), also grouped in Class II, do not encode a transposase; therefore, they exploit transposases encoded by autonomous elements to move throughout the genome [164].

## 3. Transposable Elements in Actinopterygians

The evolutionary dynamics of TEs are different in several lineages, which strongly support their pivotal role in genome evolution. The evaluation of mobile element impact on the actinopterygian genome is a fundamental step toward understanding the biodiversity of this taxon. With increasing genomic resources, a clear positive correlation between genome size and the percentage of TEs has been found in ray-finned fish [21,37,68,164,165]. Moreover, a wide range of TE amounts has been recorded in this taxon, with only 6% in the compact pufferfish genome and 55% in the zebrafish genome [165] (Figure 1).

Data published to date suggest that compared to other vertebrate genomes, class II DNA transposons are the most abundant component in most fish genomes [32,165]. Most TE superfamilies (i.e., *Gypsy*, *BEL/Pao*, ERV, *DIRS*, *Penelope*, *Rex6/Dong*, *R2*, *L1*, *RTE*, *L2*, *Rex1/Babar*, *Jockey*, *Helitron*, *Maverick*, *Zisupton*, *Tcl-Mariner*, *hAT*, *PIF-Harbinger*, *PiggyBac*, and *EnSpm*) are present in the actinopterygian genome, evidencing a higher diversity than that in other vertebrates [165]. Among them, *Tc/mariner*, *hAT*, *L1*, *L2*, and *Gypsy* are the most widespread and predominant TE superfamilies in fish genomes [31,68]. Comparing the distribution of the transposon superfamilies among the actinopterygians, the Cyprinidae family presents the highest level of TE diversity [165]. However, some organisms present a predominance of specific TE superfamilies, such as *Gypsy* in *Boleophthalmus pectinirostris*, *L2* and *RTE* in *Nothobranchius furzeri*, *Tc/mariner* in *Astyanax mexicanus*, and *hAT* in *Danio rerio* [165]. These elements have been preserved in the genomes of these organisms, and, thus, they could have had a pivotal role in their evolution. Shao and colleagues [165] proposed that the interaction between TEs and host genomes is comparable to that between organisms and their environments, explained by the Red Queen paradigm: harmful TEs are eliminated by host genomes, while beneficial TEs are instead preserved. Moreover, a critical role of *CR1* in vertebrate evolution has been reported by the same authors. The low copy number of *CR1* elements found in teleosts, contrary to primitive fishes and sarcopterygians, suggests the preservation and proliferation of these elements during the transition from water to land in tetrapods [165].

In the deeply branched nonteleost ray-finned fishes, the mobilome has been inferred from the genomes of the sturgeon *Acipenser ruthenus* and the spotted gar, *L. oculatus*. The former has a similar pattern to that observed in teleosts [166], while the latter shows a predominance of non-LTR retrotransposons [32,37]. The condition observed in spotted gar is also common to the elephant shark, *Callorhinchus milii*, and the lamprey, *Petromyzon marinus* [32]. The amount of non-LTR in bony fishes might be due to the presence of mechanisms restricting the invasion of retroelements in their genomes [31].

Another interesting feature of the ray-finned fish mobilome is the presence of more recent TE copies than those found in other vertebrate lineages. In particular, cod, stickleback, and fugu have very recent TE copies, and differences in TE activity can also be observed between species closely related to medaka and platyfish [37]. Kimura distance-based copy divergence analysis performed on 35 actinopterygians shows one or, at most, two TE amplification bursts [32,37,68,165]. These events were preceded by periods in which new elements arose through genetic mutations or where TEs invaded the host genome through horizontal transfer. Subsequently, natural selection and defense mechanisms of the host genome select beneficial mobile elements, and a period of coexistence between TEs and the host genome begins. These steps, which occurred during the history of TE activity, are associated with species radiation [148,167,168,169,170], suggesting that TEs are responsible for important evolutionary events.

*L. oculatus* is a nonteleost ray-finned fish that has not undergone further WGDs after those that have occurred at the base of vertebrates (1R and 2R WGDs). The quantitative analysis of TEs showed no differences among teleosts. This finding does not support any link between ancestral genome duplication and TE expansion in the teleost lineage [32]. The analysis of the *Salmo salar* genome revealed an expansion of DNA transposons, with a return to the diploid state after the 4R WGD [106]. The rediploidization is also achieved through the contraction of the genome associated with TE loss. This could explain the loss of *Rex3*, a teleost-specific non-LTR retroelement, absent in salmonids [61].

A positive correlation has been reported between the GC content of TEs and genomes [19]. Analyzing the GC% in the main TE groups, Class I retrotransposons, with 45.6%, are more GC-rich than Class II DNA transposons, with 40.1%; DIRS are the TEs with the highest GC content (53.8%), while the *CMC* transposons are the mobile elements with the lowest GC content (35.8%) in fish genomes. The GC-poor DNA transposons seem to be responsible for the overall GC homogenization of fish genomes.

## 4. Rex Retroelements

*Rex* retroelements are repeated elements that are widely distributed among teleost genomes and were deeply active during the evolution of this lineage [59,60,61]. Published in 1999 by Volff and his research team [59], the first report of three *reverse transcriptase* (*RT*)-carrying retrotransposons in the model fish *Xiphophorus maculatus* is attributable to the origin of the name *Rex* for this class of fish-specific retroelements.

A sequence derived from the Y chromosome of *X. maculatus* of the Rio Jamapa allowed Volff and colleagues firstly to isolate a truncated copy *Rex1-Ximj*, and then to evidence many other copies of this non-LTR retrotransposon in different teleost species, defining a second class of *Rex* retroelements named *Rex1.* Concerning their main structural features, *Rex1* non-LTRs are characterized by an *RT*, an apurinic/apyrimidinic (A/P*) site that can be located upstream or downstream of the *RT*-encoding region, and a 3′-UTR region. On the other hand, *Rex3* and *Rex6* retroelements harbor a gene encoding an endonuclease (*EN*) in addition to *RT*.

A high copy number of a novel class of *Rex* retroelements, the so-called *Rex6* elements, was further evidenced by Volff and colleagues in 2001 in the genomes of several teleosts [61]. *Rex6* is a member of the *R4* family [171] of non-LTR retrotransposons and it encodes a specific type of endonuclease, closely related to the type IIS restriction enzymes isolated from trypanosomes, nematodes, and arthropods [172]. *Rex6* elements have been found in many teleost orders: Anguilliformes [55,59], Beloniformes [60,61], Carangiformes [129], Centrarchiformes [59], Characiformes [79,82,149,173,174,175], Cichliformes [60,132,133,136,176,177,178,179,180], Cypriniformes [59], Cyprinodontiformes [59], Esociformes [60], Perciformes [60,181], Salmoniformes [60], Siluriformes [174,182,183,184], and Tetraodontiformes [60,61,134,142,185].

Although no evolutionary relationship among *Rex1*, *Rex3*, and *Rex6* has emerged to date, they are usually considered together in fluorescence in situ hybridization (FISH) studies, demonstrating their key role in karyotype evolution in fish (for review, see Carducci et al. [186]). Overall, their localization has been observed in heterochromatin at telomeric [82,129,173,183], pericentromeric, and centromeric regions [149,177,180,181,183] and in supernumerary chromosomes [79,176]. Of extreme interest is the non-negligible number of papers underlying the localization of *Rex* retroelements at the euchromatic level [129,134,179,182,184], strongly supporting the relatively high rate of gene-linkage disruption and chromosomal rearrangements in teleost genomes [149]. In general, the distribution of *Rex* retroelements in chromosomes varies considerably between teleost orders and families [186].

All the papers reviewed herein highlight the significant role of the *Rex* retroelements in the rapid evolution of teleosts, in particular, acting on karyotype and genome structure.

## 5. Endogenous Retroviruses

Retroviruses are viruses constituted by a single-stranded positive-sense RNA. After infection, a retrovirus reaches the cytoplasm of the host cell, where a reverse transcriptase (RT) converts its ssRNA into cDNA, ready to be integrated into the nuclear genome of the infected host cell. Once integrated, the provirus will exploit the nuclear machinery of the host cell to transcribe and translate its components. There are four main components of the basic toolkit of retrovirus genomes: long terminal repeats (LTRs), which carry a promoter sequence that mediates the interaction with integrase for retrovirus integration into the host cell genome; *gag* (*group-specific antigen*) genes, which encode structural protein components; *pol* (*polymerase*) genes, which enclose the RT, protease, and integrase domains; *env* (*envelope*) genes, which encode coat proteins [187]. Structurally, retroviruses differ from retrotransposons by the presence of genes encoding envelope proteins. Moreover, a characteristic hallmark that allows the identification of a past retrovirus infection is provided by the solo-LTR derived from ectopic homologous recombination between two LTRs [187]. Infection by a retrovirus may occur within a germline, leading to the generation of endogenous viral elements, the so-called endogenous retroviruses (ERVs).

ERVs are inherited through vertical transmission and consequently maintained within the host genome over millions of years [188]. Identified in all vertebrate lineages [189] and all belonging to the Retroviridae family, ERVs can be approximately grouped into three main classes based on the phylogenetic relationships between the seven exogenous retrovirus genera identified: Class I (closely related to Gammaretroviruses and Epsilonretroviruses), Class II (closely related to Betaretroviruses), and Class III (Spumavirus-like elements) [190]. Hayward and colleagues [189] have identified two further clades: human endogenous retroviruses S/L (HERVS/L)-like and snakehead fish retrovirus (SnRV)-like elements.

Naville and Volff [191] have shown that the overall ERV content in fish genomes ranges from 0.01 to 1%. In particular, epsilon-related retroviruses are the most frequent ERVs in ray-finned fishes [192]. The lowest value reported is 0.033% for *Takifugu rubripes* (with approximately 1800 insertions), and the maximum is 0.76% in *Danio rerio* (with more than 30,000 insertions) [191]. The best-studied ERV element in teleosts is Zebrafish Endogenous Retrovirus (ZFERV), isolated from zebrafish [193].

In addition to epsilon-related retroviruses and Snakehead fish retrovirus (SnRV)-like elements, endogenous foamy virus (EFV) sequences have been detected in different teleost species, including cod, platyfish, and zebrafish [194,195]. No reports of gamma or Class II elements have been described to date [189].

Whereas the evolutionary importance of ERVs, as a source of new genes [196] and, in general, as a mediator of gene expression [197,198] in catalyzing genome evolution, has been evidenced in mammals, nothing is known about the roles of ERVs in teleost evolution [191].

The complex evolutionary history of retroviruses has been recently investigated by Xu and colleagues [190]. Through an extensive genomic and phylogenetic analysis performed on species representing the main evolutionary lineages, of which 66 were ray-finned fishes, the authors unveiled the role of teleosts and turtles, as vehicles for retrovirus transmission, in overcoming the water–land barrier.

## 6. TEs and Sex Chromosomes

The wide chromosomal diversity in teleosts (e.g., interspecific diploid number variation; the presence or absence of sex and supernumerary chromosomes) has been suggested to be correlated with the ability to incorporate transposable elements [199]. The evolutionary success of TEs in a given population is strictly linked to their persistence, which is obtainable through TE vertical transmission in the germline, from one to the next generation [199]. Moreover, the accumulation of repetitive sequences is a common phenomenon in sex chromosomes, characterized by the absence of recombination [200].

Several papers have reported the involvement of *Rex* retrotransposons in the differentiation of sex chromosomes [149,173,175,181], with a key role played by *Rex6* [173]. These elements have been mapped on the sex chromosomes of four species belonging to the Characiformes [173,175] and one species of Perciformes [181] and on the largest pair of chromosomes recognized as sexual chromosomes in one species belonging to the Cichliformes [180]. Other convincing examples of the role of TEs in the control of sexual development and function have been recently reviewed by Dechaud and colleagues [199]. A clear example of TE control in a germline through *cis*-regulation was reported in the medaka, *Oryzias latipes*: a *LINE/Rex1* retroelement was found within the nonautonomous P element *Izanagi*, corresponding to the upstream region of the master sex-determining gene (*dmrt1bY*) in medaka. In particular, the *LINE/Rex1*-derived sequence located within the *Izanagi* element carries the binding site for Sox5, a transcriptional factor involved in the regulation of *dmrt1bY* [201,202]. A role of TEs in the determination of sex chromosome structure and evolution has also been observed in *X. maculatus,* in which the accumulation and spreading of *Texim* genes in only the Y chromosome is due to the activity of *Helitron* transposons, deeply influencing the evolution of this chromosome in platyfish [203]. Finally, in salmonids, analyses performed on the boundary regions of the master sex-determining gene (*sdY*) have shown a certain accumulation of TEs, which is probably responsible for the different *sdY* gene chromosomal locations despite their conservation [105,204].

## 7. Fish Transposons and the Environment

Both abiotic and biotic factors are continuously changing, resulting in new selective pressures that challenge population survival. To cope with these changes, organisms colonize new habitats and exploit their phenotypic plasticity and/or adaptive evolutionary traits. Natural selection allows organisms with features appropriate for a specific environment to survive and, thus, to reproduce, increasing their fitness. Genetic variants will be transmitted to the next generation, increasing in frequency in the population. Genetic variation can be caused by not only point mutations and whole-genome duplications but also TE activity. Moreover, transposons can be co-opted and exapted, creating regulatory sequences, coding exons, or entirely new genes useful for the host genome [22,23,37,205]. Indeed, a great number of reports have suggested the responsiveness and susceptibility of TEs to environmental changes or stressful conditions [206,207,208,209,210,211,212,213,214]. Yuan and colleagues [31] analyzed 52 fishes and reported an increase in the DNA transposons in bony fish living in freshwaters and an abundance of tandem repeats in marine species that was not explained by phylogenetic relationships. In particular, among DNA transposons, *Tc1* is the most well-represented in freshwater bony fishes. This association clearly suggests a potential role of TEs in the adaptation of fish to their living environments. Freshwater environments might encourage the proliferation and spread of DNA transposons, probably because transposition can cause new genetic variants useful for host adaptation to the environment. According to these authors, the large number of repetitive elements can contribute to the generation of novel genes useful for adaptability to the environment. Moreover, the presence of such a high content of repetitive elements can cause unstable genomes due to recombination and splicing events. Due to natural selection, uncontrollable increases in genome size do not occur. Auvinet and colleagues [148] reported a preferential accumulation of four families of *DIRS1* in specific chromosomal locations of the Antarctic teleost species belonging to the *Trematomus* genus. According to these authors, the concentration of these TEs in pericentromeric and centromeric areas could have been mediated by multiple glacial-interglacial cycles that took place in the Antarctic region. The variation in temperature probably led to changes in epigenetic regulation that have allowed TE bursts. An interesting correlation between TEs and the environmental temperature has also been evidenced by our group in a recent publication [207], in which a phylogenetic analysis was performed on the partial *reverse transcriptase* of the *Rex3* retroelement in 39 teleost species. Surprisingly, in this investigation, there was a lack of correspondence with the canonical taxonomy relationships. Indeed, the *Rex3* sequences analyzed clustered into two groups, strictly related to the environmental temperature in which these species live, suggesting a selective role of temperature on specific TE sequence variants.

## 8. Conclusions

Actinopterygii is a taxon characterized by a high diversity of species adapted to a wide range of environments. There is generally a positive correlation between genome size and TE coverage, and the major contributors to the genome size variation are DNA transposons. The data summarized here show that the ray-finned fish genomes are unique among vertebrates in their overall TE composition. The high level of TE diversity suggests that these genetic elements represent an important evolutionary tool that has had a pivotal role in fish evolution. However, it is not clear whether repetitive elements lead to environmental adaptation or vice versa [31].

Moreover, significant differences are also evident in TE activity, which might be linked to body temperature and host defense mechanisms. Indeed, body temperature is influenced by environmental conditions, which could affect the activity of the proteins involved in transposition mechanisms; the capacity to replicate and compete with other TEs is influenced by host defense mechanisms, such as piRNAs and methylation. However, information about genome size and data on genome sequencing in ray-finned fishes is still limited. Such is the case for the deep-branching nonteleost ray-finned fishes belonging to the Polypteriformes; the investigation of the genomes of these taxa could be extremely useful for providing information on the common ancestor of TEs among actinopterygian species.

## Figures and Tables

**Table 1:** Table summarizing works published to date about transposable elements in teleost orders.

Teleost Orders	References
Polypteriformes	
Acipenseriformes	[24,25]
Amiiformes	
Lepisosteiformes	[19,20,26,27,28,29,30,31,32,33,34,35]
Elopiformes	[36]
Albuliformes	
Notacanthiformes	
Anguilliformes	[24,32,36,37,38,39,40,41,42,43,44,45,46,47,48,49,50,51,52,53,54,55,56,57,58,59,60,61,62]
Osteoglossomorpha	[63,64]
Clupeiformes	[65,66]
Alepocephaliformes	
Gonorynchiformes	[67]
Cypriniformes	[14,68,69,70,71,72,73,74,75,76]
Characiformes	[77,78,79,80,81,82,83,84,85,86,87,88,89,90,91]
Gymnotiformes	[92,93,94]
Siluriformes	[14,31,95,96,97,98,99,100]
Lepidogalaxiiformes	
Argentiniformes	[101]
Galaxiiformes	[102]
Esociformes	[103]
Salmoniformes	[104,105,106,107]
Osmeriformes	[108,109]
Stomiatiformes	[109]
Ateleopodiformes	[109]
Aulopiformes	[109,110]
Myctiophiformes	[109]
Lampriformes	[109]
Percopsiformes	[109]
Zeiformes	[109]
Stylephoriformes	[109]
Gadiformes	[111,112,109]
Polymixiiformes	[109]
Trachicthtyiformes	
Beryciformes	[34,109,113,114,115,116]
Holocentriformes	[109]
Ophidiaria	[109,111,117]
Batrachoidiaria	[109]
Pelagiaria	[109]
Synghatharia	[118]
Gobiaria	[109]
Anabantaria	[109,119]
Carangaria	[109,120,121,122]
Ovalentaria	[109,123,124,125,126]
Eupercaria	[30,31,109,119,127,128,129,130,131,132,133,134,135,136,137,138,139,140,141,142,143,144,145,146,147,148,149,150,151,152]
